# Effectiveness of switching between TNF inhibitors in patients with axial spondyloarthritis: is the reason to switch relevant?

**DOI:** 10.1186/s13075-020-02288-8

**Published:** 2020-08-21

**Authors:** Santiago Rodrigues Manica, Alexandre Sepriano, Fernando Pimentel-Santos, Nélia Gouveia, Anabela Barcelos, Jaime C. Branco, Miguel Bernardes, Raquel Miriam Ferreira, Elsa Vieira-Sousa, Sofia Barreira, Filipe Vinagre, Raquel Roque, Helena Santos, Nathalie Madeira, João Rovisco, Alexandra Daniel, Sofia Ramiro

**Affiliations:** 1 Department of Rheumatology, Hospital Egas Moniz, Centro Hospitalar Lisboa Ocidental, EPE, R. da Junqueira 126, 1349-019 Lisbon, Portugal; 2 CEDOC, NOVA Medical School, Universidade de Lisboa, Lisbon, Portugal; 3 Leiden University Medical Center, Leiden, The Netherlands; 4 Department of Rheumatology, Centro Hospitalar Baixo Vouga, Aveiro, Portugal; 5 iBimed, University of Aveiro, Aveiro, Portugal; 6 Department of Rheumatology, Centro Hospitalar de São João, Porto, Portugal; 7 Department of Medicine, Faculty of Medicine, University of Porto, Porto, Portugal; 8 Rheumatology and Metabolic Bone Diseases, Hospital de Santa Maria - Centro Hospitalar Universitário Lisboa Norte, EPE, Lisbon, Portugal; 9 Rheumatology Research Unit, Instituto de Medicina Molecular - Faculty of Medicine, Lisbon Academic Medical Centre, University of Lisbon, Lisbon, Portugal; 10 Rheumatology, Hospital Garcia de Orta, Almada, Portugal; 11 Rheumatology, Instituto Português de Reumatologia, Lisbon, Portugal; 12 Centro Hospitalar e Universitário de Coimbra, Coimbra, Portugal; 13 Rheumatology Department, Centro Hospitalar de Leiria, Leiria, Portugal; 14 Department of Rheumatology, Leiden University Medical Center, Leiden, The Netherlands; 15 Department of Rheumatology, Zuyderland Medical Center, Heerlen, The Netherlands

**Keywords:** Treatment, bDMARD, TNFi, axSpA, AS, r-axSpA, nr-axSpA, Switch, Ankylosing spondylitis, Spondyloarthritis

## Abstract

**Background:**

To investigate whether the reason to discontinue the first TNF inhibitor (TNFi) affects the response to the second TNFi in axial spondyloarthritis (axSpA).

**Methods:**

Patients with axSpA from the Rheumatic Diseases Portuguese Register (ReumaPt), who discontinued their first TNFi and started the second TNFi between June 2008 and May 2018, were included. Response was assessed by the Ankylosing Spondylitis Disease Activity Score (ASDAS) clinically important improvement (ASDAS-CII), major important improvement (ASDAS-MI), low disease activity (ASDAS-LDA), and inactive disease (ASDAS-ID). The reason for discontinuation of the first TNFi was defined, according to ASDAS-CII as primary failure (no response ≤ 6 months), secondary failure (response ≤ 6 months but lost thereafter), adverse events, and others. The association between the reason for discontinuation of the first TNFi and response to the second TNFi over time was assessed in multivariable generalized equation (GEE) models.

**Results:**

In total, 193 patients were included. The reason for discontinuation of the first TNFi did not influence the response to the second TNFi, according to the ASDAS-CII. However, a difference was found with more stringent outcomes, e.g., there was a higher likelihood to achieve ASDAS-ID with the second TNFi for patients discontinuing the first TNFi due to secondary failure (OR 7.3 [95%CI 1.9; 27.7]), adverse events (OR 9.1 [2.5; 33.3]), or other reasons (OR 7.7 [1.6; 37.9]) compared to primary failure.

**Conclusion:**

Patients with axSpA with secondary failure to their first TNFi, compared to those with primary failure, have a better response to the second TNFi according to stringent outcomes.

## Background

Tumor necrosis factor inhibitors (TNFi) have revolutionized the treatment of patients with axial spondyloarthritis (axSpA). Nevertheless, some patients discontinue TNFi [1] either because of inefficacy or safety reasons. The number of discontinuations varies across studies but can go up to 50–60% in 2 years [2]. In such circumstances, the practicing clinician has to decide what to offer as a second-line therapy, considering the available treatment options.

For many years, TNFi were the only biological disease-modifying drugs (bDMARD) with proved efficacy in axSpA. More recently, secukinumab and ixekizumab, both IL-17 inhibitors (IL-17i), have demonstrated efficacy in phase 3 randomized controlled trials (RCTs) [3–5], both in TNFi-naive and TNFi-experienced patients with radiographic axial spondyloarthritis (r-axSpA). Part of these data already translated into a change in the 2016 update of the Assessment of SpondyloArthritis International Society–European League Against Rheumatism (ASAS-EULAR) management recommendations for axSpA [6] which prescribes switching to another TNFi or an IL-17i in case of failure of the first TNFi.

In 2019, the American College of Rheumatology/Spondylitis Association of America/Spondyloarthritis Research and Treatment Network (ACR/SAA/SPARTAN) 2019 update on the treatment recommendations for axSpA [7] prioritized an IL-17i over a second TNFi in patients with primary non-response to the first TNFi and a second TNFi over an IL-17i for those with secondary non-response (after 6 months). This recommendation reflects the long-lasting hypothesis that the reason for discontinuing the first TNFi affects the response to the second TNFi. However, evidence to support such hypothesis is still scarce. In the absence of RCTs to address this clinically relevant issue, clinicians have relied on data stemming from observational studies yielding conflicting results, thus precluding definitive conclusions [2, 8–12].

The aims of this study were twofold: to compare the efficacy of TNFi as the first-line and second-line therapy and to assess whether the reason for discontinuation of the first TNFi affects the response to the second TNFi in patients with axSpA.

## Methods

### Patients and study design

In this prospective multicenter cohort study, adult patients with axSpA, according to their treating rheumatologists, registered in Reuma.pt (Rheumatic Diseases Portuguese Register) who discontinued their first TNFi (for any reason) and started a second TNFi between June 2008 and May 2018, were included. Patients were required to have complete data on Ankylosing Spondylitis Disease Activity Score (ASDAS) at baseline and 3 and 6 months after starting the first TNFi. Patients were assessed at baseline, 3 months, 6 months, and then every 6 months, up to 10 years of follow-up. Data was collected by the treating rheumatologist and stored centrally in the Reuma.pt online database. Reuma.pt is a nationwide clinical register, established and managed by the Portuguese Society of Rheumatology, in which data from patients with various rheumatic diseases is recorded. Since 2008, patients have been included in the register, with regular information on their visits to the rheumatologist. First patients with inflammatory arthritis including rheumatoid arthritis, axSpA, psoriatic arthritis, and juvenile idiopathic arthritis on biological treatment were included, and later, the register was expanded to other treatments and also to other diseases. A detailed report of the design of Reuma.pt and data management procedures has been published elsewhere [13].

For the current study, a dedicated team of researchers from each participating center was assigned to compare information on a core set of variables between the central database and the medical records, in order to complete missing information whenever possible.

Reuma.pt has been approved by the ethics committees of the participating hospitals and complies with the Declaration of Helsinki. This specific study has been approved by the ethics committee of the NOVA Medical School, Lisbon, Portugal. Patients have signed a written informed consent before inclusion.

### Outcomes

The main outcome was the ASDAS clinically important improvement (ASDAS-CII), defined as a decrease from baseline of ≥ 1.1 units of the ASDAS score. Secondary outcomes were as follows: ASDAS major improvement (ASDAS-MI), defined as a decrease from baseline of ≥ 2.0 units; ASDAS low disease activity (ASDAS-LDA), i.e., an ASDAS < 2.1 [14]; ASDAS inactive disease (ASDAS-ID), i.e., ASDAS < 1.3; and the Bath Ankylosing Spondylitis Disease Activity Index 50 (BASDAI50), defined as 50% improvement compared to baseline.

### Reason to discontinue TNFi

The reason for discontinuation of the first TNFi was defined as follows: (i) primary failure, if ASDAS-CII was not achieved at 3 or 6 months; (ii) Secondary failure, if ASDAS-CII was achieved at 3 or 6 months but lost in ≥ 1 subsequent visit; (iii) adverse event; and (iv) others (e.g., pregnancy, surgery). Primary and secondary failure were defined a posteriori by computing the response according to the ASDAS-CII in patients with reported discontinuation due to the lack of efficacy by the treating rheumatologist. For patients with ≥ 1 reason for discontinuation recorded by their treating rheumatologist, the “main” reason was defined in a case-by-case decision basis. For instance, if a patient experienced a severe adverse event by the time of discontinuation, this was selected as the main reason even if ASDAS-CII had not been achieved.

An alternative definition of secondary failure to the first TNFi was used in a sensitivity analysis. A patient was considered to have a secondary failure if he/she experienced a flare (ASAS definition, 0.9 point increase in ASDAS between two consecutive time points) [15], comparing to the immediately preceding visit (or to 2 previous visits, if the preceding was missing for ASDAS), at least once, after achieving ASDAS-CII at 3 or 6 months.

### Statistical analysis

Baseline characteristics were described, at the start of the second TNFi, for the entire population and compared according to the reason for discontinuation of the first TNFi using analysis of variance (ANOVA) for continuous variables and the *χ*^2^ test for categorical variables.

The proportion of patients meeting the efficacy outcomes, at 3 and 6 months, was assessed both for the first and second TNFi (in patients with complete data for each outcome and per time point), adjusting for potential confounders selected a priori on clinical grounds (age, gender, and C-reactive protein (CRP) levels at baseline) in logistic regression models.

The association between the reason for discontinuation of the first TNFi and response to the second TNFi during up to 10 years of follow-up was tested in binomial generalized estimating equation (GEE) models, to take into account the within-patient correlation across repeated measures of the outcome over time. All models were adjusted for age, gender, and CRP, the latter modeled as time-varying. A sensitivity analysis was conducted in the same way. *p* values lower than 0.05 were considered significant. Data analysis was performed using Stata version 14.0.

## Results

### Patient characteristics

In total, 346 patients with axSpA registered in Reuma.pt by the time of database lock (May 2018) had discontinued the first TNFi and started the second TNFi; out of these, 193 had available data for ASDAS at baseline, 3 and 6 months for the first TNFi, and were therefore included. Patients had a mean age of 45 years, 53% were male, 61% were HLA-B27-positive, had a mean follow-up of 1.5 years (range 3 months to 10 years), and 149 (88%) had radiographic axSpA. Patients eligible for the study were largely similar to those excluded (Online Supplementary Table S1).

### Patients’ characteristics according to the reason for discontinuation of the first TNFi

Most baseline characteristics were similar across the reason for discontinuation of the first TNFi, including co-medication (non-steroidal anti-inflammatory drugs (NSAIDs), glucocorticoids, and conventional synthesis disease-modifying drugs (csDMARD)) (Online Supplementary Table S2). The proportion of male patients was higher among patients who discontinued their first TNFi due to secondary failure (60%) and adverse events (59%) than among patients with primary failure (25%) and other reasons for discontinuation (41%).

Only 31% of patients with primary failure had an elevated CRP (≥ 0.5 mg/dL) at baseline. This percentage was higher for secondary failure, adverse events, and other reasons, 70%, 55%, and 53%, respectively.

### Comparison of response between the first and second TNFi

In total, 96 patients had data for ASDAS for the first and second TNFi at baseline and 3 months and 79 at baseline and 6 months. The adjusted response rate to the second TNFi was lower compared to the response to the first TNFi (Table 1). For some outcomes, the difference was large, e.g., ASDAS-CII at 3 months (41% vs 51%) and 6 months (35% vs 56%) and ASDAS-MII at 3 months (13% vs 33%) and 6 months (22% vs 32%).

**Table 1:** Efficacy of the first and second TNFi (adjusted models)

	1st TNF % (95% CI)	2nd TNF % (95% CI)
**Outcome at 3 M defined (*n*** = 96*)		
**ASDAS CII**	51 (42; 60)	41 (31; 50)
**BASDAI50**	45 (36; 54)	39 (29; 48)
**ASDAS MII**	33 (25; 42)	13 (6; 19)
**ASDAS LDA**	42 (32; 51)	26 (18; 35)
**ASDAS inactive**	19 (12; 26)	16 (9; 22)
**Outcome at 6 M defined (*n*** = 79*)		
**ASDAS CII**	56 (46; 66)	35 (26; 45)
**BASDAI50**	46 (35; 56)	33 (23; 43)
**ASDAS MII**	32 (22; 41)	22 (13; 30)
**ASDAS LDA**	41 (30; 51)	29 (19; 39)
**ASDAS inactive**	11 (5; 18)	15 (8; 22)

Percentages and 95% CI are derived from logistic regression models adjusted for age, gender, and CRP at baseline

*ASDAS* Ankylosing Spondylitis Disease Activity Score, *BASDAI* Bath Ankylosing Spondylitis Activity Index, *LDA* low disease activity, *ID* inactive disease, *CII* clinically important improvement, *MII* major important improvement, *BASDAI50* Bath Ankylosing Spondylitis Disease Activity Index 50, *TNFi* tumor necrosis factor inhibitor, *M* months

*96 patients had ASDAS and BASDAI outcomes available at baseline and 3 months for both TNFi; 79 patients had ASDAS and BASDAI outcomes available at baseline and 6 months for both TNFi

### Association between reason for discontinuation of the first TNFi and efficacy of the second TNFi

There was no association between the reason to discontinue the first TNFi and response to the second TNFi as defined by ASDAS-CII. Such association could be found with the most stringent outcomes, namely ASDAS-MI and ASDAS-ID (Table 2). For instance, compared to patients who discontinued their first TNFi due to primary failure, patients were more likely to achieve ASDAS-ID with the second TNFi if they discontinued their first TNFi due to secondary failure (OR 7.3 [95% CI 1.9; 27.7]), adverse events (OR 9.1 [2.5; 33.3]), or other reasons (OR 7.7 [1.6; 37.9]).

**Table 2:** Association between the reason for discontinuation of the first TNFi and response to the second TNFi over time

Reason to discontinue the first TNFi*	Outcome for the second TNFi OR (95% CI)				
	ASDAS-CII (***n*** = 135)	ASDAS-MII (***n*** = 135)	ASDAS-LDA (***n*** = 166)	ASDAS-ID (***n*** = 166)	BASDAI50 (***n*** = 147)
**(Ref. primary failure)**					
**-Secondary failure**	1.9 (0.7; 4.8)	**4.8 (1.3; 18.2)**	1.2 (0.6; 2.4)	**7.3 (1.9; 27.7)**	1.4 (0.6; 3.0)
**-Adverse events**	1.5 (0.6; 3.5)	2.4 (0.6; 9.6)	0.9 (0.5; 1.7)	**9.1 (2.5; 33.3)**	1.1 (0.5; 2.3)
**-Others**	1.0 (0.3; 3.8)	1.7 (0.1; 19.4)	1.0 (0.4; 2.4)	**7.7 (1.6; 37.9)**	0.5 (0.1; 1.7)

*ASDAS* Ankylosing Spondylitis Disease Activity Score, *LDA* low disease activity, *ID* inactive disease, *CII* clinically important improvement, *MII* major important improvement, *BASDAI50* Bath Ankylosing Spondylitis Disease Activity Index 50, *N* number

*Generalized estimated equation (GEE) models with the reason for discontinuation of the first tumor necrosis factor inhibitor (TNFi) as a predictor (reference category: primary failure); all models adjusted for age, gender, and C-reactive protein. Models include all visits during follow-up of up to 10 years. Odds ratios (OR) in bold are statistically significant (*p* < 0.05)

When considering the alternative definition of secondary failure (i.e., according to ASAS Flare), results were mostly similar. However, patients who discontinued their first TNFi due to secondary failure were also more likely to achieve ASDAS-CII with the second TNFi (OR 3.0 [95%CI 1.1; 8.5]) compared to those who had primary failure to their first TNFi (Online Supplementary Table S3).

## Discussion

In this prospective observational cohort study, we have compared the response to a second TNFi according to the reason for discontinuation of the first TNFi in patients with axSpA using longitudinal data. Response was better for patients discontinuing the first TNFi due to secondary failure compared to those with primary failure. These results can help the practicing clinician to decide on which drug, or drug class, to use as a second-line therapy in daily practice in patients with axSpA.

We found a lower response to the second TNFi compared to the first TNFi. In a Finnish cohort study with 543 patients, the BASDAI50 response after 6 months was achieved by 52% and 25% of the patients with their first and second TNFi treatments, respectively [12]. Retention of the second TNFi was shorter than of the first TNFi. In a French study with 244 patients (101 under the first TNFi and 143 on their second TNFi), the drug survival for the first and second TNFi was 22 and 15 months, respectively (*p* < 0.01), regardless of the individual drug [16]. Concerning ASDAS outcomes, as observed in our study, in a Swiss study, ASDAS-ESR inactive disease state was reached by 4% of patients after previous primary failure against the 22% after secondary failure [2].

Compared to patients with primary failure, those with secondary failure to the first TNFi had a better response to the second TNFi, but only for the most stringent outcomes (ASDAS-MI and ASDAS-ID). This may explain the previous negative results. Studies in which the subtle association between the reason for discontinuation of the first TNFi and efficacy of the second TNFi was not found using BASDAI [9–11], or ASAS40 as efficacy outcomes [9], which are far less strict than ASDAS-ID. Differences in the population and also in the methods used to define primary and secondary failure may also contribute to explain the differences across studies. In our study, patients with a primary failure to the first TNFi had, at baseline of the second TNFi, lower levels of CRP and were also most likely female, which are known to associate with worse response to TNFi treatment [17, 18]. However, a worse response was still seen despite adjustment for these characteristics.

Our study is not without limitations. First, residual confounding can still explain the differences found between patients discontinuing the first TNFi due to primary and secondary failure. The number of confounders that we could adjust for was also limited by the small sample size. However, residual confounding is an inherent problem in observational research, and arguably, we have adjusted for the most relevant confounders. Secondly, we could not compare the efficacy of starting a second TNFi or an IL-17i, after failing the first TNFi due to small numbers of patients treated with IL-17i. Additionally, no information was available on non-pharmacological interventions, which are the first line in axSpA. Our data suggest that it could be better to choose a drug with a mechanism of action other than TNFi in case of primary failure to a TNFi, and future studies should give resolution whether that is the case for IL-17i.

## Conclusions

In summary, on average, response to a second TNFi is worse than the response to a first TNFi, especially among patients that never responded in the first place to this class of drugs.

## Supplementary information

**Additional file 1 **: **Online supplementary Table S1**. Baseline patient- and disease- characteristics at the start of the second TNFi in the entire population. **Online supplementary Table S2**. Baseline patient- and disease- characteristics at the start of the second TNFi in the entire population and per reason of discontinuation of the first TNFi. **Online Supplementary Table S3**. Association between the reason for discontinuation of the first TNFi and response to the second TNFi (with alternative definition of secondary failure to first TNFi).

## Data Availability

The data that support the findings of this study are available from Reuma.pt (Portuguese Society of Rheumatology) but restrictions apply to the availability of these data, which were used under license for the current study, and so are not publicly available. Data are however available from the authors upon reasonable request and with permission from Reuma.pt Scientific Committee.
