# Supplementary material for: Effectiveness of switching between TNF inhibitors in patients with axial spondyloarthritis: is the reason to switch relevant?
Source: Arthritis Res Ther. 2020 Aug 21;22:195. doi: 10.1186/s13075-020-02288-8 (PMC7441644; doi:10.1186/s13075-020-02288-8)
Supplement: Supplementary file 1 — Additional file 1 : Online supplementary Table S1. Baseline patient- and disease- characteristics at the start of the second TNFi in the entire population. Online supplementary Table S2. Baseline patient- and disease- characteristics at the start of the second TNFi in the entire population and per reason of discontinuation of the first TNFi. Online Supplementary Table S3. Association between the reason for discontinuation of the first TNFi and response to the second TNFi (with alternative definition of secondary failure to first TNFi). [file 13075_2020_2288_MOESM1_ESM.docx]

**Online supplementary table S1 – Baseline patient- and disease- characteristics at the start of the second TNFi in the entire population**

| Variables | Total  (n=346) | Included  (n=193) | Excluded  (n=153) | p-value |
| --- | --- | --- | --- | --- |
| Age** | 45 (11) | 45 (11) | 45 (11) | 0.90 |
| Symptom duration (years)** | 5 (11) | 14 (10) | 3 (8) | 0.05 |
| Months between 1^st^ and 2^nd^ TNFi | 5 (11) | 6 (13) | 3 (8) (n=149) | 0.05 |
| Gender (male) | 179 (52) | 103 (53) | 77 (50) | 0.64 |
| mNY** | 149 (88) | 149 (88) (n=170) | 103 (83) (n=124) | 0.30 |
| HLA-B27** | 180 (57) | 113 (61) (n=184) | 67 (51) (n=131) | 0.07 |
| Peripheral arthritis** | 144 (46) (n=315) | 81 (44) (n=184) | 63 (48) (n=131) | 0.48 |
| Enthesitis** | 98 (31) (n=315) | 54 (29) (n=184) | 44 (34) (n=131) | 0.42 |
| Uveitis** | 78 (25) (n=315) | 51 (28) (n=184) | 27 (21) (n=131) | 0.15 |
| Dactylitis** | 13 (4) (n=315) | 5 (3) (n=184) | 8 (6) | 0.14 |
| IBD ** | 25 (8) (n=315) | 14 (8) (n=184) | 11 (8) (n=131) | 0.80 |
| Elevated CRP#** | 177 (56) | 101 (55) (n=184) | 76 (58) | 0.58 |
| First TNFi, n(%)  -Adalimumab  -Etanercept  -Golimumab  -Infliximab | 83 (24)  92 (27)  51 (15)  120 (35) | 59 (31)  46 (24)  18 (9)  70 (36) | 24 (16)  46 (30)  33 (22)  50 (33) | **<0.01** |
| Second TNFi  -Adalimumab  -Certolizumab-Pegol  -Etanercept  -Golimumab  -Infliximab | 111 (32)  13 (4)  127 (37)  55 (16)  40 (12) | 55 (29)  5 (3)  80 (41)  30 (16)  23 (12**)** | 56 (37)  8 (5)  47 (31)  25 (16)  17 (11) | 0.19 |
| BASDAI (0-10)** | 5.2 (2.4) (n=282) | 5.0 (2,5) | 5.5 (2.3) | 0.07 |
| ASDAS** | 3.2 (1.2) | 3.2 (1.2) | 3.4 (1.1) (n=100) | 0.34 |
| CRP (mg/dL)** | 1.9 (3.5) (n=265) | 1.8 (2.7) | 2.2 (4.4) (n=112) | 0.33 |
| Co-medication**  -NSAIDs  -csDMARDs  -Steroids | (n=343)  124 (36)  121 (35)  54 (16) | (n=191)  76 (40)  74 (39)  33 (17) | (n=152)  48 (32)  47 (31)  21 (14) | 0.12  0.13  0.38 |

**<15% missing data; All ASAS features assessed as recommended by the ASAS (20); #Defined as CRP≥0.5mg/dL; p-value assessed ANOVA test for continuous variables and χ^2^ test for discrete variables. For continuous variables mean (SD) are shown while for discrete variables total frequency (n) and relative frequency (%) are shown. TNFi: Tumour necrosis factor inhibitor; mNY: modified New York criteria; HLA-B27: human leucocyte antigen B27; ASAS: assessment of spondyloarthritis international society; IBD: Inflammatory bowel disease; CRP: C-reactive protein; BASDAI: Bath Ankylosing Spondylitis Disease Activity Index; ASDAS: Ankylosing Spondylitis Disease Activity Score; BASFI: Bath Ankylosing Spondylitis Functional Index; BASMI: Bath Ankylosing Spondylitis Metrology Index; NSAID: Non-steroidal anti-inflammatory drug; csDMARD: common synthesis disease modifying anti-rheumatic drug.

**Online supplementary table S2 – Baseline patient- and disease- characteristics at the start of the second TNFi in the entire population and per reason of discontinuation of the first TNFi**

| Variables | Total  (n=193) | Reason to discontinue 1^st^ TNFi | | | | |  |
| --- | --- | --- | --- | --- | --- | --- | --- |
|  |  | **Primary failure**  **(n=26;13%)** | **Secondary failure**  **(n=48;25%)** | **Adverse events**  **(n=102;53%)** | **Other reason**  **(n=17;9%)** | **p-value** |  |
| Age** | 45 (11) | 46 (10) | 45 (10) | 44 (11) | 44 (15) | 0.95 |  |
| Symptom duration (years)** | 14 (10) | 16 (11) | 17 (11) | 17 (11) | 21 (13) | 0.15 |  |
| Months between 1^st^ and 2^nd^ TNFi | 6 (13) | 4 (10) | 3 (4) | 7 (16) | 9 (16) | 0.13 |  |
| Gender (male) | 103 (53) | 6 (25) | 29 (60) | 60 (59) | 7 (41) | **<0.01** |  |
| mNY** | 149 (88) | 19 (79) | 40 (91) | 76 (87) | 14 (93) | 048 |  |
| HLA-B27** | 113 (61) | 11 (42) | 33 (72) | 61 (64) | 8 (47) | 0.05 |  |
| Peripheral arthritis** | 81 (44) | 7 (27) | 22 (48) | 45 (47) | 7 (41) | 0.28 |  |
| Enthesitis** | 54 (29) | 9 (35) | 14 (30) | 31 (33) | 0 (0) | 0.05 |  |
| Uveitis** | 51 (28) | 5 (19) | 12 (26) | 27 (28) | 7 (41) | 0.47 |  |
| Dactylitis** | 5 (3) | 1 (4) | 1 (2) | 3 (3) | 0 (0) | 0.87 |  |
| IBD ** | 14 (8) | 0 (0) | 5 (11) | 7 (7) | 2 (12) | 0.35 |  |
| Elevated CRP#** | 101 (55) | 8 (31) | 32 (70) | 52 (55) | 9 (53) | **0.02** |  |
| First TNFi, n(%)  -Adalimumab  -Etanercept  -Golimumab  -Infliximab | 59 (31)  46 (24)  18 (9)  70 (36) | 7 (27)  3 (12)  10 (38)  6 (32) | 19 (40)  12 (25)  2 (4)  15 (31) | 32 (31)  22 (22)  3 (3)  45 (44) | 1 (6)  9 (53)  3 (18)  4 (24) | **<0.01** |  |
| Second TNFi  -Adalimumab  -Certolizumab-Pegol  -Etanercept  -Golimumab  -Infliximab | 55 (29)  5 (3)  80 (41)  30 (16)  23 (12**)** | 5 (19)  2 (8)  14 (54)  1 (4)  4 (15) | 16 (33)  0 (0)  18 (38)  8 (17)  6 (13) | 24 (24)  1 (1)  46 (45)  20 (20)  11 (11) | 10 (59)  2 (12)  2 (12)  1 (6)  2 (12) | **<0.01** |  |
| BASDAI (0-10)** | 5.0 (2,5) | 6.2 (1.7) | 5.2 (2.2) | 4.9 (2.7) | 3.1 (1.9) | **0.02** |  |
| ASDAS** | 3.2 (1.2) | 3.4 (1.0) | 3.5 (1.0) | 3.2 (1.3) | 2.3 (1.1) | **0.02** |  |
| CRP (mg/dL)** | 1.8 (2.7) | 1.0 (1.1) | 2.5 (3.4) | 1.6 (2.3) | 1.6 (4.1) | 0.11 |  |
| BASFI (0-10) | 5.0 (2.7)  (n=156) | 5.1 (2.4)  (n=26) | 5.3 (2.3)  (n=44) | 4.9 (3.0)  (n=73) | 3.7 (2.7)  (n=12) | 0.35 |  |
| Co-medication**  -NSAIDs  -csDMARDs  -Steroids | 76 (40)  74 (39)  33 (17) | 10 (38)  15 (58)  4 (15) | 24 (50)  20 (42)  8 (17) | 39 (39)  34 (34)  20 (20) | 3 (18)  5 (29)  1 (6) | 0.13  0.13  0.54 |  |

**<15% missing data; All ASAS features assessed as recommended by the ASAS (20); #Defined as CRP≥0.5mg/dL; p-value assessed ANOVA test for continuous variables and χ^2^ test for discrete variables. For continuous variables mean (SD) are shown while for discrete variables total frequency (n) and relative frequency (%) are shown. TNFi: Tumour necrosis factor inhibitor; mNY: modified New York criteria; HLA-B27: human leucocyte antigen B27; ASAS: assessment of spondyloarthritis international society; IBD: Inflammatory bowel disease; CRP: C-reactive protein; BASDAI: Bath Ankylosing Spondylitis Disease Activity Index; ASDAS: Ankylosing Spondylitis Disease Activity Score; BASFI: Bath Ankylosing Spondylitis Functional Index; BASMI: Bath Ankylosing Spondylitis Metrology Index; NSAID: Non-steroidal anti-inflammatory drug; csDMARD: common synthesis disease modifying anti-rheumatic drug.

Patients who discontinued their first TNFi due to other reason (n=17) had the followed registered cause; Pregnancy (planning), n=4; Uveitis under TNFi, n=7, sarcoidosis diagnosis, n=1; surgery, n=3 and inflammatory bowel disease (IBD) onset under TNFi, n=3.

**Online Supplementary Table S3** - Association between the reason for discontinuation of the first TNFi and response to the second TNFi (with alternative definition of secondary failure to first TNFi)

|  | Outcome for the second TNFi  OR (95% CI) | | | | |
| --- | --- | --- | --- | --- | --- |
| Reason to discontinue first TNFi*  (ref Primary failure)  -Secondary failure  -Adverse events  -Other | **ASDAS-CII**  **(N=123)** | **ASDAS-MII**  **(N=123)** | **ASDAS-LDA**  **(N=151)** | **ASDAS-ID (N=151)** | **BASDAI50**  **(N=133)** |
|  | **3.0 (1.1;8.5)**  1.4 (0.5;3.4)  0.8 (0.2;3.1) | **7.3 (1.9;28.0)**  2.5 (0.7;9.2)  1.6 (0.2;16.8) | 1.2 (0.6;2.6)  0.9 (0.5;1.7)  1.0 (0.4;2.4) | **8.9 (2.3;34.9)**  **9.3 (2.6;33.4)**  **7.7 (1.6;36.9)** | 2.0 (0.9;4.6)  1.2 (0.6;2.7)  0.5 (0.1;1.7) |

*Generalised estimated equation (GEE) models with the reason of discontinuation of the first tumour necrosis factor inhibitor (TNFi) as predictor (reference category: primary failure); all models adjusted for age, gender and C-reactive protein. Odd Ratios (OR) in bold are statistically significant (p<0.05). ASDAS: The Ankylosing Spondylitis Disease Activity Score; LDA: Low disease activity; ID: Inactive disease; CII: Clinically important improvement; MII: Major important improvement; BASDAI50: Bath Ankylosing Spondylitis Disease Activity Index 50.
